# On the interpretation of the atmospheric mechanism transporting the environmental trigger of Kawasaki Disease

**DOI:** 10.1371/journal.pone.0226402

**Published:** 2019-12-16

**Authors:** Joan Ballester, Sílvia Borràs, Roger Curcoll, Albert Navarro-Gallinad, Sofya Pozdniakova, Lidia Cañas, Jane C. Burns, Xavier Rodó

**Affiliations:** 1 Climate and Health Program (CLIMA), Barcelona Institute for Global Health (ISGlobal), Barcelona, Catalonia, Spain; 2 Institute of Environmental Sciences and Technologies (ICTA), Autonomous University of Barcelona (UAB), Barcelona, Catalonia, Spain; 3 Kawasaki Disease Research Center, University of California San Diego (UCSD), La Jolla, California, United States; 4 Catalan Institution for Research and Advanced Studies (ICREA), Barcelona, Catalonia, Spain; Columbia University, UNITED STATES

## Abstract

Recent advances on the environmental determinants of Kawasaki Disease have pointed to the important role of the atmospheric transport of a still unknown agent potentially triggering the disease. The hypothesis arose from an innovative methodology combining expertise in climate dynamics, the analysis of ocean and atmosphere data, the use of dispersion models and the search for biological agents in air samples. The approach offered a new perspective to reveal the identity of the potential trigger, but at the same time, it increased the level of complexity, which could potentially lead to the misinterpretation of the mechanisms. Some years after it was originally formulated, we here provide a brief clarification on the approach and limits of the methodology in order to prevent an eventual misuse of our research ideas and theory, so that further research can better focus on the knowledge gaps that still remain open.

## Introduction

The etiology of Kawasaki Disease (KD) remains unknown after five decades of active research. Recent studies have analyzed the potential connections between KD and a diversity of environmental factors and mechanisms, and among them, important advances have pointed to the relevant role of the atmospheric transport of a still unknown agent triggering the disease. This line of research explored the connections between the temporal components of KD in Japan (i.e. the country with the largest incidence) and nearby sites with those arising from the complex dynamics of tropospheric winds. As such, these analyses mounted on early investigations showing a pronounced seasonality of the disease in Japan, with peaks in late winter and early spring and a trough in the fall [1]. More recently, Rodó et al. [2] revealed a consistent atmospheric circulation bridge across the mid-latitude Pacific Ocean explaining the major epidemics in Japan, as well as the seasonality and major non-epidemic interannual fluctuations in Japan and San Diego. Ballester et al. [3] later showed how these fluctuations are controlled by a larger-scale, coupled ocean-atmosphere climatic phenomenon, the El Niño-Southern Oscillation, which is predictable two seasons in advance. Finally, Rodó et al. [4] pointed to possible microbial antigens or toxins in aerosol particles as a possible trigger for KD, which are capable of eliciting an idiosyncratic immune response in susceptible children. This pathway, though not proved, would be consistent with an agricultural source, a short incubation time and synchronized outbreaks [5].

Based on these results, Manlhiot and colleagues [6] recently analyzed 8 years of data from all pediatric hospital admissions with a primary or secondary diagnosis of KD in Canada (except Quebec) to assess whether periods of high disease numbers were related to an environmental trigger that is transported by tropospheric winds from China and Japan, as originally demonstrated in Rodó et al. [2]. For that purpose, in Manlhiot et al. [6], the seasonality in KD was not filtered out; instead they smoothed and standardized the time series and composited the surface winds for the set of 15 winter (14 summer) months above (below) the +1 (-1) standard deviation level. Although they recognize that the study cannot infer causality, and even acknowledged the potentially speculative nature of some of the mechanisms and associations, the authors found that westerly winds over the mid-latitude Pacific Ocean were stronger during months with high numbers of KD cases in Canada. They stated that this association might be consistent with the long-range atmospheric transport of a potential trigger for KD from eastern Asia.

Beyond the strict analysis and interpretation of the work by Dr. Manlhiot and colleagues, the present short letter is aimed at clarifying some possible misunderstandings arising from the methodology used to analyze the atmospheric pathway, as well as to provide a wider context to better interpret the potential limits of the mechanism transporting the still unknown agent potentially triggering the disease.

## Methods

**Fig 1:**
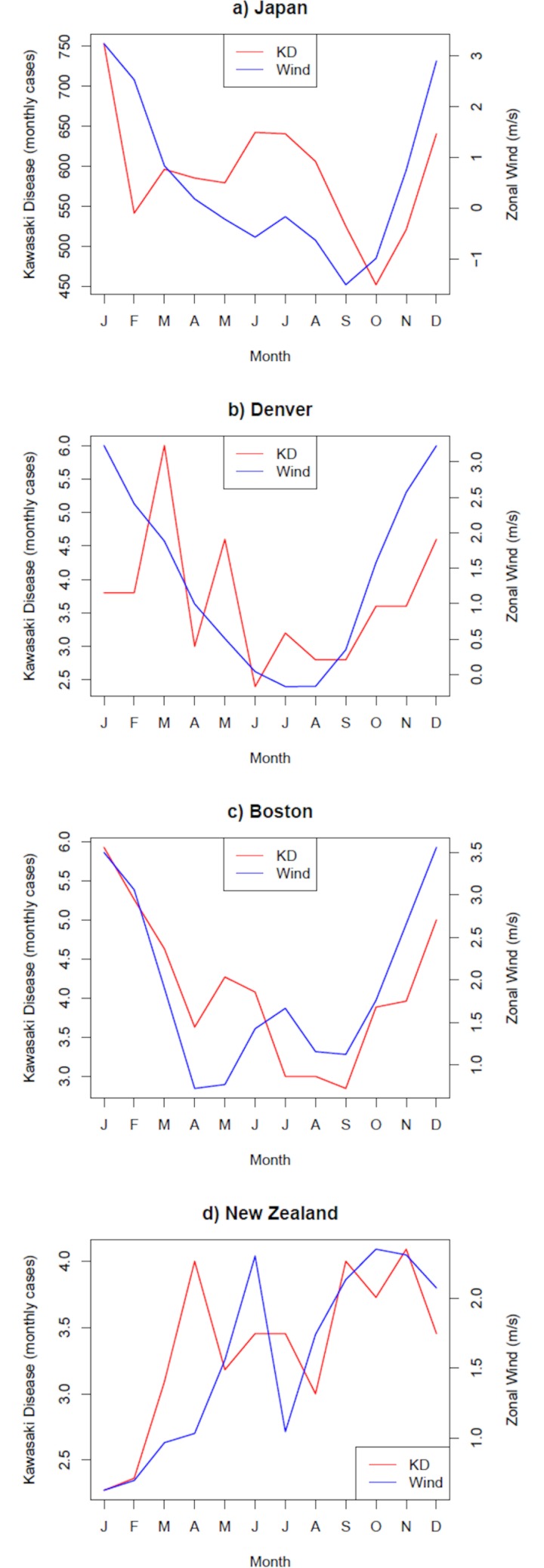
Seasonality of Kawasaki Disease (red, monthly cases) and the zonal component (positive means eastward) of surface winds (blue, m/s).

**Fig 2:**
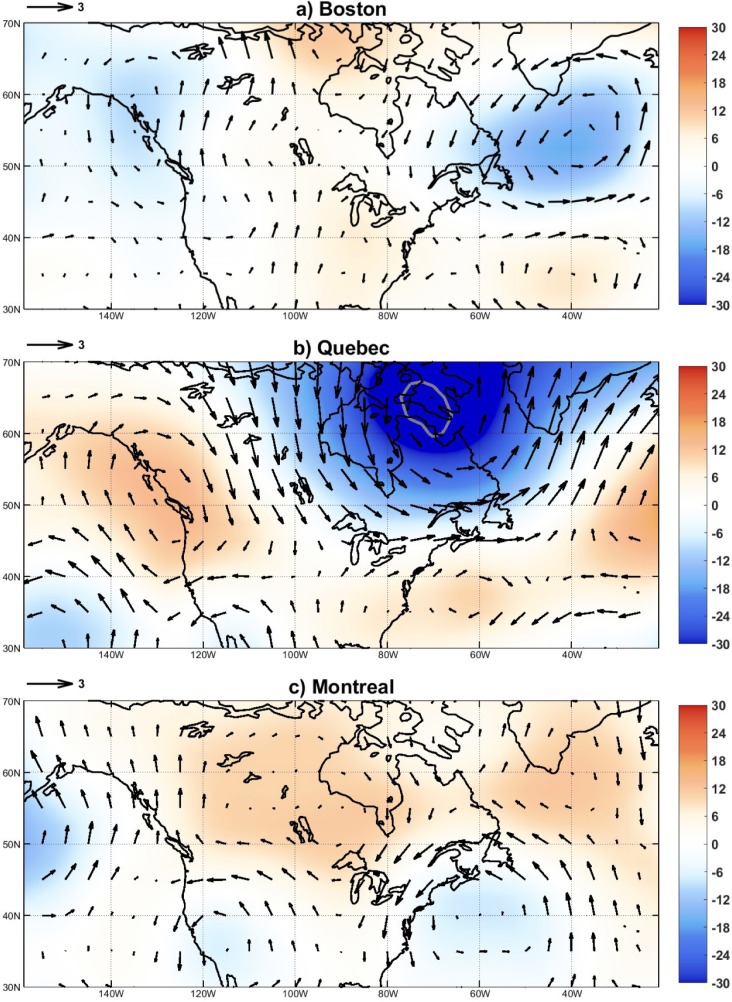
Composites of geopotential height (shading, in m) and wind (arrows, m/s) anomalies at 300 hPa for the interannual peaks of Kawasaki Disease in Boston (a), Montreal (b) and Quebec (c). Peaks were chosen, and composites computed, according to the methodology in Ballester et al. [3]. Grey contours depict shaded areas with significant anomalies at the p < 0.05 level.

KD data for Japan (1979–2016, N = 344032 cases), Denver (2004–2008, N = 221 cases), Boston (1983–2009, N = 1306 cases), Quebec (1985–2007, N = 419 cases), Montreal (1985–2008, N = 824 cases) and New Zealand (1995–2006, N = 441 cases) was obtained through the Kawasaki Disease Global Climate Consortium [7]. Atmospheric data was derived from the NCEP/NCAR reanalysis [8]. The seasonality in Fig 1 was computed as the mean annual cycle for both KD and zonal winds. The method to compute the interannual composites in Fig 2 is strictly the same than in Ballester et al. [3]: a recursive Butterworth filter with cutoff period of 18 months was used to calculate the interannual component of detrended variables, which was then standardized, i.e. the mean was removed and the difference was divided by the standard deviation. The composites show the standardized interannual component of atmospheric variables for subset of peaks reaching the +1 standard deviation criterion in the interannual KD time series.

## Results

We would like to express our caution on the treatment of climate data to test the airborne nature of the KD trigger, and in general, for any climate-related disease. KD in most of the northern mid-latitude sites is seasonal, with maximum incidence generally around winter (red curves in Fig 1A–1C). KD is also seasonal in the only mid-latitude site of the southern hemisphere for which data is available (New Zealand), although the maximum is observed later in spring (Fig 1D). From a climate perspective, both the northern and southern mid-latitudes are characterized by the seasonal reinforcement and meridional displacement of the prevailing westerlies, and particularly the jet streams in the upper troposphere (see for example Supplementary Fig 1 in Ballester et al. [3]). This synchronicity explains why it is relatively easy to find associations between the seasonality of climate variables and the seasonality of climate-related diseases, such as the one described in Manlhiot and colleagues, or those shown in Fig 1A–1D.

To effectively circumvent this problem and remove the seasonality effect, in previous studies we checked the consistency of the mechanism explaining the atmospheric transport of the environmental trigger through an analysis of the major epidemics in Japan and the interannual component of KD in Japan and San Diego [2,3]. Our main hypothesis was that if such a mechanism exists, demonstrating that it operates at different timescales would strongly support it whilst explaining the different frequency components of the disease. Otherwise, an alternative mechanism effectively driving the variability of the disease at some, but not all, temporal scales should be argued, which albeit theoretically possible, would be much more difficult to find and demonstrate.

During these investigations, we successfully found and characterized a unique mechanism explaining the seasonality, epidemics and interannual fluctuations of KD across the mid-latitude Pacific Ocean through the respective component in the tropospheric winds. We explored other nearby sites, mainly in eastern Asia and North America, and tried to explain the interannual component of KD through the same large-scale atmospheric transport mechanism. The search was, however, unsuccessful for some locations. In the particular case of Canada and the northern United States, we considered the relatively long records of Boston, Quebec and Montreal, for which we could similarly extract an interannual component with enough peaks to calculate composite averages (this is not possible for relatively short time series). Results, which were not documented at the time, show very different and generally not significant wind patterns, suggesting that the direct effect of the atmospheric bridge across the mid-latitude Pacific Ocean is not controlling, at least, the year-to-year variability of the disease in eastern North America (Fig 2). Against the idea of a unique global source affecting the disease worldwide, this hypothesis could be compatible with the existence of other more local sources of the air-borne agent carried by winds, for example linked to agricultural activities, with a much limited radius of influence [4].

All these considerations make us extremely cautious regarding the model framework for the global distribution of KD proposed by Manlhiot and colleagues (see Table 4 therein), which uses incidence estimates from all countries with published reviews and abstracts, and not only the few sites described in Rodó et al. [2] and subsequent papers. In Manlhiot's model, the eastward and southward distances from eastern Asia (100°E, 60°N) along the zonal and meridional axes are considered and stated as significant variables explaining the global distribution of KD cases. The existence of an atmospheric pathway linking only a few sites on both shores of the mid-latitude Pacific Ocean, and not further east, however, seems to rule out the hypothesis of a centralized origin that explains a fraction of the incidence of the disease worldwide. A global pathway connecting the northern mid-latitudes with the tropics and the southern hemisphere and controlling the variability of the disease, at any temporal scale, would be even more unlikely given the complex dynamics of the climate system and tropospheric winds.

## Concluding remark

Recent advances on the environmental factors of the disease were generated within the framework of a new line of research triggered by an innovative methodology combining expertise in climate dynamics, the analysis of ocean and atmosphere data, the use of dispersion models and the search for biological agents in air samples. The approach opened a new perspective to the analysis of the mechanisms, but at the same time, it added a new level of complexity that could potentially lead to the misinterpretation of the results. The air-borne hypothesis was carefully validated by checking the consistency of atmospheric dynamics across temporal scales and through different modelling techniques. With this perspective in mind, we would like to express a note of caution on the potential misuse and misinterpretation of the atmospheric mechanisms described in our previous work, so that further research is better focused on current knowledge gaps. New avenues will most likely come from the use of alternative epidemiological techniques to analyze other temporal scales of the wind-disease association, the analysis of additional tropospheric variables associated with wind variability, and especially, a more systematic sampling of the biological content of air masses through new laboratory techniques.
